# Poor Quality Embryos Hamper the Development of High Quality Ones

**Published:** 2017

**Authors:** Mohammad Reza Sadeghi

Embryo quality and embryo selection are critical criteria for embryo transfer and subsequently success of assisted reproductive technologies (ART). Transfer of good quality embryos is associated with increased implantation, pregnancy and live birth rates and also decreased pregnancy loss and prenatal complications in comparison with transfer of embryos with impaired quality. Furthermore, advance in embryo culture techniques and embryo selection criteria and technologies lead to a decrease in number of transferred embryos without significant decline in ART success. Therefore, elective single embryo transfer (*eSET*) is recently the dominant discourse in the community and infertility clinics. An important concern of eSET is reduced multiple pregnancy and its prenatal complications as a major *complication* of ART. Since more than one embryo is produced in IVF cycle, surplus embryos with appropriate quality are frozen for future transfer in frozen/thawed cycles. There are treatment cycles in which the number and quality of embryos are not sufficient for freezing/thawing. The question always arises in these cycles is that “Does simultaneous transfer of low and top quality embryos have determining impact on treatment success or not?”. It was previously believed and recent finding reported that the low quality embryos have no impact on implantation and development of top quality embryos; even, many low quality embryos have the potential for implantation and successful pregnancy. As a result, twin pregnancy is 9.5% in cycles with transfer of one top quality and one low quality embryo; however, it is zero in eSET and 10.6% in cycles with two top quality embryos (1).

Although other studies provide different conclusions, the transfer of low quality embryos can impair implantation and development of top quality embryos. So, it is recommended to transfer top quality embryos alone despite the minimum number of fresh/frozen embryos in transfer cycles (2). If this is true, the question arises that how poor-quality embryos influence development and implantation of the embryos with good quality. Review of *in vitro* studies on prolonged culture of embryos confirms the *in vivo* findings. Group culture of day 3 good and low quality embryos influences the rate of high quality blastocyst formation in comparison with separate culture of low and high quality embryos. It has been postulated that different toxic compounds such as ammonia and reactive oxygen species (ROS) produced by low quality embryos have deleterious effects on quantity and quality of developed blastocysts (3). In addition, endometrial epithelial cells (EECs) and stromal cells (*ESCs*) of *uterine* tissues are very responsive to human embryos following *in vitro* culture. Top quality embryos enhance diffrentiation, decidualization, secretion of chemokine and growth factors that promote encapsulation and implatation of blastocyst. Therefore, good quality embryos actively intract with endometrium and promote their immplantation. In contrast, poor quality embryos inhibit selective migratory response of decidualizing ESCs and subsequent active encapsulation of themselves (4). Thus, *in vitro* culture of embryo and the co-culture with *ESCs verified the recent report of* El-danasouri *et al. on negative effects of poor quality embryos on implantation of good quality ones in tranfer cycles.* Of course, it also should not be ignored that embryo assessments are now based on morphometric criteria which are not *accurate* and *precise* parameters. Hence, in some cases, this assessment is not consistent with functional markers of embryos. The support for this claim are the many reports on successful pregnancy following transfer of poor quality embryos or simultaneous transfer of one top and one poor quality embryo in one cycle and the incidence of having twins is 9.5% among cases (1).

In spite of the supporting evidence for influence of embryo quality on the implantation, it should be noted that all of these studies have been retrospective. The final confirmation of these findings requires numerous prospective studies with more stringent design on larger samples. Regarding partial verification of this issue, IVF centers should avoid simultaneous transfer of good and poor quality embryos in fresh and frozen/thawed cycles as much as possible. It is recommended that poor quality embryos be cultured separately and subsequently transferred or frozen if they could develop to high quality blastocysts.
